# Visual Parameters and Retinal Morphology for Polypoidal Choroidal Vasculopathy Pre- and Post-Intravitreal Ranibizumab with or without Photodynamic Therapy: A Short-Term Prospective Study

**DOI:** 10.3390/ijerph18052581

**Published:** 2021-03-04

**Authors:** Rituparna Ghoshal, Sharanjeet Sharanjeet-Kaur, Norliza Mohamad Fadzil, Somnath Ghosh, Nor Fariza Ngah, Roslin Azni Binti Abd Aziz

**Affiliations:** 1 Optometry & Vision Science Program, Faculty of Health Sciences, Universiti Kebangsaan Malaysia, Jalan Raja Muda Abdul Aziz, Kuala Lumpur 50300, Malaysia; rituparna4ab@yahoo.co.in (R.G.); norlizafadzil@ukm.edu.my (N.M.F.); 2 Department of Allied Health Sciences, Brainware University, Barasat, Kalkata, West Bengal 700125, India; somnath4ab@yahoo.co.in; 3 Department of Ophthalmology, Hospital Shah Alam, Persiaran Kayangan, Seksyen 7, Shah Alam 40000, Malaysia; drfarizangah@gmail.com (N.F.N.); roslinazni@gmail.com (R.A.B.A.A.)

**Keywords:** combination therapy, polypoidal choroidal vasculopathy, retinal morphology, visual functions

## Abstract

The objective of this study was to compare visual parameters and retinal layers’ morphology pre-treatment (baseline) and 6 months post-treatment in polypoidal choroidal vasculopathy (PCV) eyes. A single centre, longitudinal, prospective study was conducted at a public tertiary hospital of Malaysia. Visual parameters including distance and near visual acuity (DVA and NVA), contrast sensitivity (CS), reading speed (RS), and different qualitative and quantitative optical coherence tomography (OCT) parameters were evaluated pre- and 6 months post-treatment. Thirty-three naïve PCV eyes of 32 patients (mean age of 67.62 years) were evaluated pre- and post-treatment of intravitreal ranibizumab with and without photodynamic therapy. After treatment, sub retinal fluid decreased from 27 eyes (84.35%) at baseline to 7 eyes (21.88%) at 6 months while pigment epithelium detachment decreased from 32 eyes (100%) at base line to 15 eyes (46.87%) at 6 months. Mean pre-treatment quantitative morphological OCT retinal parameters including thickness and volume of central sub field, center thickness, center minimum, and maximum thickness reduced significantly. Similarly, all visual parameters including DVA, NVA, CS, and RS showed statistically significant improvement. While 89% of the eyes showed improvement in CS, 78%, 71%, and 65% of the eyes showed improvement in NVA, RS, and DVA, respectively. Thus, CS was the most treatment responsive visual parameter.

## 1. Introduction

Polypoidal choroidal vasculopathy (PCV) is a commonly seen subtype of Asian neovascular age-related macular degeneration (n-AMD) [1,2,3,4,5,6]. Clinical features of PCV eyes have been described in previous studies [7,8,9]. Though the existence of PCV came to notice almost three decades ago [10], a unanimous treatment guideline of PCV is yet to be established. Many of the clinical trials reported a combined therapy of intravitreal anti-vascular endothelial growth factor (anti-VEGF) injection and photodynamic therapy (PDT) as the most promising treatment option, followed by PDT monotherapy and anti-VEGF monotherapy [11,12,13,14,15]. A recent study from an AMD referral centre of Malaysia showed a similar approach in treating PCV with combined therapy being the most frequently used treatment approach [16]. However, treatment outcomes of Malaysian PCV eyes has not yet been published. Furthermore, in eyes with typical n-AMD, visual functions including contrast sensitivity, reading speed, and near visual acuity have been reported to present more robust information than a single distance visual acuity measurement [17]. Although the morphological changes in PCV have been documented well [8,9], the visual parameters apart from distance visual acuity after treatment of PCV eyes have not been studied well [11,12,13,14]. Like in other macular pathologies, a comprehensive evaluation of different visual parameters including distance and near visual acuity, contrast sensitivity, and reading speed pre- and post-treatment is likely to provide complete understanding of visual outcome in the PCV eyes. Thus, the objective of this study was to evaluate and compare the visual parameters and retinal morphology pre-treatment and 6 months post-treatment in PCV eyes.

## 2. Materials and Methods

### 2.1. Study Design and Participants

This was a longitudinal prospective study, conducted in an ophthalmology clinic of an AMD referral public hospital of Malaysia. Patients clinically diagnosed with PCV in at least one eye by a senior retinal consultant (R.A.A.A.) were recruited. Then patients with naïve PCV who were advised to undergo intravitreal ranibizumab injection with or without PDT were included in this study. The diagnosis of PCV has been described in our earlier paper [9]. Briefly, the Japanese study criteria for PCV diagnosis was used where findings of polyps and/or a branch vascular network on indocyanine green angiography (ICGA) is considered as a gold standard for PCV diagnosis [18]. However, when patients had allergy to indocyanine, ICGA was not performed, but optical coherence tomography (OCT) parameter features such as sharp retinal pigment epithelium detachment (RPED) peak, double-layer sign, multiple retinal pigment epithelium detachment (RPED), RPED notch, hyporeflective lumen representing polyps, and hyper-reflective intraretinal hard exudates were taken into consideration. The first two features and at least one of the other features sufficed for the diagnosis of PCV. In the absence of the first two features, the diagnosis of PCV was also made when at least three of the other features were present simultaneously [19]. Patients with any retinal pathology other than PCV, such as other types of AMD, diabetic retinopathy, retinal vein occlusion, central serous retinopathy, or a macular hole; any history of treatment (anti-vascular endothelial growth factor injection, photodynamic therapy, or laser) for PCV; and cognitive, hearing, or motion impairment were excluded from the study.

### 2.2. Sample Size Determination

Formula for comparing two groups’ means (dependent groups) was used to calculate the sample size [20].

$$n=2+C\;{\left[\frac{s}{d}\right]}^{2}=2+7.8{\left[\frac{0.435}{0.27}\right]}^{2}=22.24$$

where

n = sample size,

C = dependent on the value chosen on significance level (α) and power (1 − β)

S = standard deviation

D = difference researcher would like to detect.

The parameters were set as follows: α = 5%, power of the study = 80%, d = 0.27, which was difference of mean best corrected distance visual acuity pre- and post-anti-VEGF treatment and pooled standard deviation of parameter tested (with mean BCVA pre anti-VEGF 0.57 ± 0.48 and post anti-VEGF treatment of 0.30 ± 0.39) = 0.435 [21]. The value for C = 7.8 with 80% power and 5% alpha.

Therefore, a sample size of 22 was determined. With a dropout chance of 20%, the sample size was set at minimum of 26.

Ethics approval was obtained from the Universiti Kebangsaan Malaysia Research and Ethics Committee (UKM 1.5.3.5/244/NN-186-2014) and the Medical Research and Ethics Committee of National Medical Research Register (NMRR-16-1965-31826), Ministry of Health, Malaysia, which follows the tenets of the Declaration of Helsinki. Written informed consent was obtained from all participants.

### 2.3. Visual Parameters

All examinations were conducted at baseline prior to commencement of treatment and after 6 months of treatment. A 4-m early treatment diabetic retinopathy study (ETDRS) chart was used to measure the best corrected distance visual acuity (DVA). Each letter that was identified correctly was assigned a value of 0.02 log MAR. Pelli-Robson chart was used at 1 m to measure contrast sensitivity (CS). Near visual acuity (NVA) and reading speed (RS) were recorded using a UiTM Malay related word reading chart [22]. NVA was recorded as the threshold or the smallest print size that was read correctly by the patient with best spectacle correction whereas RS was recorded by dividing the number of words that were read correctly with the time taken to read in words per minute.

### 2.4. Retinal Morphology

Spectral Domain OCT (Spectralis HRA+OCT Heidelberg Engineering Inc., Heidelberg, Germany) was used to evaluate the retinal morphology of the study eyes. Pre-set qualitative and quantitative analysis of OCT images at 1 mm center of the fovea was performed by two examiners (RAAA and RG) at different points of time in different orders in a masked fashion. Qualitative OCT parameters included presence of pigment epithelium detachment (PED), sub retinal fluid (SRF), sub retinal tissue (SRT), intra retinal fluids (IRF), and polyp. Integrity of junction of inner segment and outer segment of photoreceptor (IS-OS junction), external limiting membrane (ELM), and retinal pigment epithelium-Bruch’s membrane (RPE-BM) complex were also assessed. Furthermore, quantitative parameters including mean retinal thickness and volume of central 1 mm of fovea (RT and RV), central thickness (CT), center maximum, and center minimum thickness (CTmax and CTmin) were measured in OCT thickness map using the incorporated software of Spectralis OCT.

### 2.5. Image Analysis

The image analysis has been described in detail in our previous paper [9]. IS-OS, ELM and RPE-BM were graded as present when 75% or more of the parameters were seen, discontinuous when less than 75% but more than 25% of the parameters were seen, and absent when 25% or less were seen [9].

### 2.6. Treatment of the Study Eyes

All patients included in this study received a first dose of intravitreal anti-VEGF 0.5 mg ranibizumab (Lucentis; Genentech, South San Francisco, CA, USA) at baseline. This was usually combined with first sitting of photodynamic therapy (PDT) with verteporfin. Location and size of the PCV lesion was evaluated by ICGA. Subsequently, the study eye received anti-VEGF injection at month 2 and 3. However, PDT was deferred in eyes with poor vision, larger lesion, multiple polyps foveal fibrosis, and thinned fovea as in these instances PDT is not the preferred choice of treatment. For example, treating multiple polyps with a single beam of PDT or managing a large lesion with PDT could be quite difficult [23,24].

### 2.7. Statistical Analysis

All the data were analyzed using SPSS software IMB SPSS 17; SPSS Inc., Chicago, IL, USA. Paired *t*-test, Wilcoxon sign rank test, and McNemar test were employed to compare the visual and morphological parameters at baseline and 6 months follow up. A two-tailed probability of 0.05 or less was considered as statistically significant.

## 3. Results

Thirty-two eyes of 31 patients with newly diagnosed PCV undergoing treatment for PCV were followed up for a period of six months. Demographic details of the patients have been described in Table 1. The majority of the eyes (n = 26) underwent combination therapy with ranibizumab and PDT at 1st month of diagnosis and ranibizumab at 2nd and 3rd month followed by another dose of ranibizumab as and when advised by the clinician. The rest of the six eyes underwent anti-VEGF monotherapy with ranibizumab. Average number of anti-VEGF injection at six months was 3.44.

### 3.1. Visual Parameters at Baseline and after 6 Months

**Figure 1:**
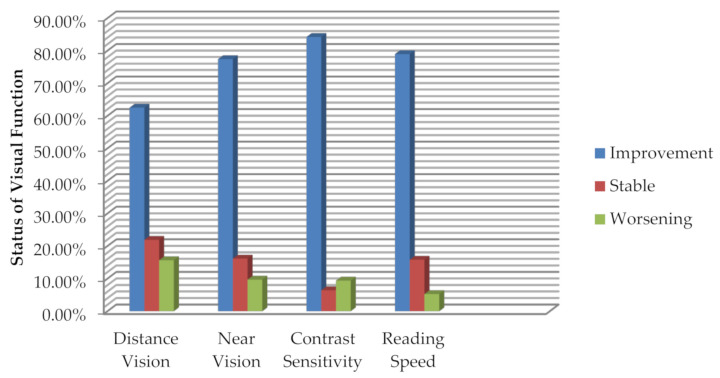
Status of distance visual acuity, near visual acuity, contrast sensitivity after 6 months: Improvement = 0.1 logMAR or more improvement; Stable = less than 0.1 logMAR change; Worsening = 0.1 logMAR or worse. Status of reading speed after 6 months: Improvement = 10 words per minute or more improvement; Stable = less than 10 words per minute change; Worsening = 10 words per minute or worse.

Visual parameters including distance visual acuity (DVA), near visual acuity (NVA), contrast sensitivity (CS), and reading speed (RS) showed statistically significant improvement (*p* ≤ 0.05) after six months of follow up in PCV eyes after treatment (Table 2). Twenty eyes showed improvement of 0.1 logMAR (5 letters) or more in DVA whereas 24 eyes showed improvement of 0.1 logMAR or more in NVA and 27 eyes showed improvement of 0.15 log CS (one triplet) or more in CS. Furthermore, RS improved by 10 or more words per minute in 15 eyes (Figure 1).

### 3.2. Morphology of Study Eyes Pre-Treatment and 6 Months Post-Treatment

Exudative changes decreased noticeably with subretinal fluid (SRF) and pigment epithelium detachment (PED) being in 27 eyes (84.35%), and 32 eyes (100%) from pre-treatment to 7 eyes (21.87%) and 15 eyes (46.87%), respectively, 6 months post-treatment.

Integrity of external limiting membrane (ELM) at foveal 1000 micron significantly improved after 6 months (*p* = 0.048) with 62.5% of the eyes showing ELM present at 75% or more area of foveal 1000 µ. Although the integrity of inner segment and outer segment of photoreceptor junction (IS-OS junction) and retinal pigment epithelium-Bruch’s membrane complex (RPE-BM complex) improved after treatment, the difference was not statistically significant (*p* = 0.369 and 0.180 respectively).

**Figure 2:**
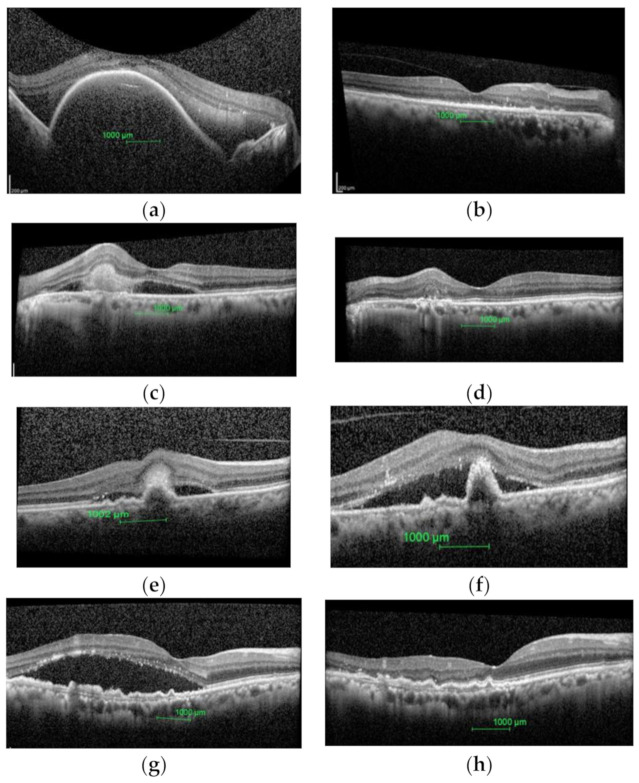
Pre-treatment and post-treatment images of polypoidal choroidal vasculopathy (PCV) eyes obtained by SD-OCT. (**a**) Pre-treatment PCV eye with large PED and hypo reflective area attached to it. (**b**) Post-treatment with no PED. (**c**) Pre-treatment PCV eye with SRF and sub-retinal hemorrhage. (**d**) Post-treatment with no SRF and resolved sub-retinal hemorrhage. (**e**) Pre-treatment PCV eye with multiple PED with SRF and sub-retinal hemorrhage. (**f**) Post-treatment with SRF, multiple PED with medium reflective areas attached to the central PED. (**g**) Pre-treatment PCV eye with SRF and multiple hyper reflective spots. (**h**) Post-treatment with no SRF and few hyper reflective spots.

Table 3 shows a detailed description of all the qualitative parameters at pre-treatment and 6 months post-treatment in the study eyes. Figure 2 shows OCT findings of PCV eyes at pre-treatment and 6 months post-treatment. Similarly, all quantitative morphological OCT parameters used in the present study showed significant improvement (*p* < 0.05).

## 4. Discussion

The majority of the PCV patients (n = 26) in the present study underwent combination therapy with ranibizumab and PDT. This is in correspondence with the previous research that reported combined PDT and ranibizumab is the most commonly used treatment option in PCV patients seen in a tertiary hospital of Malaysia [16]. Anti-VEGF therapy alone has shown limited efficacy in polyp regression which can be more efficiently treated with PDT [13,25]. PDT is well recognized for the treatment of PCV through ‘thrombosis’ of the abnormal vessels. Thus, a combination therapy of anti-VEGF and PDT is considered as superior in achieving better morphological and visual outcome in PCV eyes [13]. However, in eyes with large PCV lesions, multiple polyps, and poorer baseline visual acuity, PDT has failed to gain any added visual enhancement over anti-VEGF monotherapy [26]. Similarly, in the present study, combination therapy was first choice of treatment in naive PCV eyes followed by ranibizumab monotherapy in eyes where PDT was deferred.

In the present study, all the quantitative morphological parameters including the mean total retinal thickness (RT), mean total retinal volume (RV), center thickness (CT), maximum thickness of central 1 mm ETDRS grid (CTmax), and minimum thickness of central 1 mm ETDRS grid (CTmin) improved significantly (*p* ≤ 0.05) 6 months post-treatment, indicating morphological improvement of the study parameters. Similarly, exudative changes had also decreased noticeably with sub retinal fluid (SRF) and pigment epithelium detachment (PED) being present in 84.35% and 100% of the eyes at base line (pre-treatment) and 21.87% and 46.87% of the eyes 6 months post-treatment. This is similar to the previous findings that reported improvement of both quantitative and qualitative retinal morphological parameters after the treatment in PCV [13,27]. Like typical neovascular AMD, in PCV eyes, vascular endothelium growth factor is reported to be increased causing exudative changes in retina [28]. Intravitreal anti-VEGF injections exhibit good treatment outcome in PCV eyes by reducing the SRF and other exudative changes and thereby normalizing retinal thickness.

Furthermore, in the present study, integrity of external limiting membrane (ELM) had improved noticeably. Sharanjeet-Kaur et al. [9] have reported integrity of ELM as an important parameter that represents visual functions in eyes with PCV. A damaged ELM disrupts normal connections between the photoreceptor and Muller cells which results in the structural and functional dysfunction of photoreceptors. Coscus et al. [29] have reported restoration of ELM after anti-VEGF in eyes with neovascular AMD. In this context, the present study is the first to report reconstruction of ELM after 6 months of anti-VEGF in PCV eyes. However, integrity of IS-OS junction and RPE-BM complex did not show statistically significant change after 6 months. This is in contrast to the findings of Coscus et al. [29] who reported significant improvement of IS-OS junction disruption 18 months after anti-VEGF in neovascular AMD eyes. Nevertheless, the assessment method of the two studies was different. While, the present study used a qualitative approach towards measuring IS-OS junction at 1000 micron of fovea, Coscus et al. [29] measured the length of IS-OS junction at foveal 2000 micron using software. Furthermore, the present study reports 6 months outcome of study eyes. A long-term follow-up of the study eyes will further enhance our understanding on the same.

Another major aim of the study was to report comprehensive visual parameters pre- and post-treatment in PCV eyes. Keeping correspondence to the morphological improvement, all the visual parameters in the present study including mean DVA, NVA, CS, and RS improved significantly (*p* ≤ 0.05). This could be because the lesion in PCV initially lies below the neurosensory retina causing less visual damage. Besides, PCV is not frequently linked to fibrous proliferation causing profound vision loss which occurs in end stage typical neovascular AMD [2,30].

Although many previous studies have reported high contrast DVA post treatment in PCV eyes [13,24,27], none of them reported other visual parameters in PCV eyes. Thus, the present study is the first to report post treatment CS, NVA, and RS in PCV eyes. While 61.5% of the eyes showed improved DVA, CS showed maximum improvement with 84% of the eyes exhibiting improved contrast sensitivity from pre-treatment value. Similarly, Nixon et al. [31] reported improvement of CS when the neovascular AMD patients were shifted to aflibercept from ranibizumab, although visual acuity was stable in these patients. In the present study, the Pelli-Robson chart was used to measure CS. While the Pelli-Robson chart measures contrast at low spatial frequencies (between 0.5 to 1 cycle per degree), ETDRS high contrast acuity chart measures spatial vision at high frequency. Thus, high contrast visual acuity chart is used to assess the high spatial frequency end of the contrast sensitivity curve. A combination of Pelli–Robson CS and standard DVA thereby provides an indication of both ends of the CS curve [32].

There are previous researches indicating that CS in eyes with typical neovascular AMD sometimes provide more useful information compared to DVA alone [17,31]. Sharanjeet-Kaur et al. [9] reported CS as the most related visual parameter with the OCT parameters of treatment naive PCV eyes. Similarly, in the present study, number of eyes showing improvement in CS was more than the eyes with improved DVA, suggesting that in some cases, CS is more responsive to the treatment in PCV eyes. Similarly, NVA and RS showed improvement in 77.41% and 78% of the eyes, which is again more than 61.5% of the eyes that showed improved DVA. NVA and RS require more complex performance of eyes involving not only the foveal region but the surrounding area while high contrast DVA is more of a function of central fovea. Thus, in PCV eyes, a non-improved DVA alone may not always indicate the poor visual outcome. Measures of CS, NVA, and RS may provide further information on macular integrity in these eyes. Besides, CS, NVA, and RS are thought to be closely related to various daily living activities [33,34]. Thus, improved CS, NVA, and RS score is expected to positively impact those daily living activities. This information is worth emphasizing as it will assist clinicians and researchers in assessing and defining treatment outcomes of PCV eye.

The major strength of the study is being the first to report post treatment comprehensive visual parameters and outer retinal parameters in PCV eyes. However, a shorter study period could be considered as one of the limitations of the study. A long term follow up would be necessary to monitor the morphological and visual improvements of the study patients in the long run. Another constrain of the present study could be the inability to quantify ELM, IS-OS junction, and RPE-BM complex integrity. Some of the previous researchers have used different methods to quantify these parameters and analyzed them pre- and post-treatment in eyes with nAMD to present a more extensive report on these parameters [21,29,35]. Thus, quantification of these retinal parameters in the PCV eyes might further enhance our understanding on the same.

## 5. Conclusions

The present study was the first to evaluate and compare comprehensive visual parameters in PCV eye pre- and post-treatment. This study was also the first to report the treatment outcome of PCV eyes in Malaysian population. A majority of the study patients underwent combination therapy with ranibizumab and PDT. While exudative changes of the study eyes including SRF, IRF, PED, and presence of polyps were noticeably decreased after 6 months, integrity of ELM improved significantly. Similarly, 89% of the eyes showed improvement in CS followed by 78%, 71%, and 65% of the eyes showing improvement in NVA, RS, and DVA respectively. Thus, CS appeared as the most treatment responsive visual parameter. Therefore, the present study, apart from reporting a significant improvement in morphological and visual parameters in eye with PCV after 6 months of combination therapy, also strengthens the fact that CS along with other visual parameters should be tested to represent the treatment outcome in PCV eyes.

## Figures and Tables

**Table 1:** Demographic details of the study subjects.

Parameters	Total Number of Subjects (n = 31)
Age (mean ± Standard deviation)	67.62 ± 7.8 years
Gender	Number (%)
Male	18 (58.06)
Female	13 (41.94)
Race	
Malay	12 (38.70)
Chinese	15 (48.38)
India	4 (12.90)

**Table 2:** Evaluation of visual and retinal morphology parameters between pre-treatment and 6 months post-treatment.

Variable	Pre-Treatment	6 Months Post-Treatment	*p*
DVA	0.75 ± 0.27	0.49 ± 0.28	<0.001
NVA	0.76 ± 0.28	0.46 ± 0.265	<0.001
CS	0.76 ± 0.26	1.10 ± 0.30	<0.001
RS	59.84 ± 23.18	84.66 ± 26.15	0.001
RT	389.13 ± 114.36	283.07 ± 96.13	<0.001
RV	0.33 ± 0.13	0.22 ± 0.06	0.001
CT	373.56 ± 146.03	250.58 ± 125.90	<0.001
CTmax	486.70 ± 203.58	379.82 ± 102.39	<0.001
CTmin	310.99 ± 102.18	210.26 ± 109.48	<0.001

DVA: Distance visual acuity. NVA: Near visual acuity. CS: Contrast sensitivity. RS: Reading speed. RT: Mean total retinal thickness. RV: Mean total retinal volume. CT: Central thickness. CTmax: Maximum thickness of central 1 mm ETDRS grid. CTmin: Minimum thickness of central 1 mm ETDRS grid. *p*: *p*-values are statistically significant (*p* < 0.05).

**Table 3:** Qualitative parameters comparison between pre-treatment and 6 months post-treatment.

Qualitative Parameters	Number of Eyes at Pre-Treatment (%)	Number of Eyes at 6 Months Post-Treatment (%)	*p*
PED	32 (100%)	15 (46.87%)	<0.001
SRF	27 (84.37%)	7 (21.88%)	<0.001
IRF	6 (18.75%)	1 (3.12%)	0.032
SRT	3 (9.38%)	3 (9.38%)	0.500
Polyp	32 (100%)	12 (37.50%)	<0.001
ELM			
Present	11	20	0.048
Discontinuous	4	5	
Absent	17	7	
IS-OS junction			
Present	11	12	0.369
Discontinuous	7	12	
Absent	14	8	
RPE-BM complex			
Present	9	15	0.180
Absent	7	5	
Discontinuous	16	12	

PED: pigment epithelium detachment. SRF: sub-retinal fluid. IRF: intraretinal fluid. SRT: sub-retinal tissue. ELM: external limiting membrane. IS-OS junction: inner segment and outer segment of photoreceptor junction. RPE-BM complex: retinal pigment epithelium-Bruch’s membrane complex. *p*: *p*-values are statistically significant (*p* < 0.05).

## Data Availability

Not applicable.
